# Association between serum vitamin D3 levels and gingival crevicular fluid osteocalcin and N-terminal telopeptide levels in periodontal health and disease

**DOI:** 10.1590/1678-7765-2026-0009

**Published:** 2026-08-03

**Authors:** Burcin Cevik, Atanur Sarioglu, Tugrul Kirtiloglu, Bahattin Avci, Leman Tomak

**Affiliations:** 1 Atakum Oral and Dental Health Center Samsun Turkey Atakum Oral and Dental Health Center, Samsun, Turkey.; 2 Ondokuz Mayis University Faculty of Dentistry Department of Periodontology Samsun Turkey Ondokuz Mayis University, Faculty of Dentistry, Department of Periodontology, Samsun, Turkey.; 3 Ondokuz Mayis University Faculty of Medicine Department of Medical Biochemistry Samsun Turkey Ondokuz Mayis University, Faculty of Medicine, Department of Medical Biochemistry, Samsun, Turkey.; 4 Ondokuz Mayis University Faculty of Medicine Department of Biostatistics and Medical Informatics Samsun Turkey Ondokuz Mayis University, Faculty of Medicine, Department of Biostatistics and Medical Informatics, Samsun, Turkey.

**Keywords:** Vitamin D3, Gingival crevicular fluid, Osteocalcin, N-terminal telopeptide

## Abstract

**Objectives:**

This study aimed to investigate the association between serum vitamin D3 levels and gingival crevicular fluid (GCF) osteocalcin and N-terminal telopeptide (NTx) levels in periodontally healthy patients and those living with periodontitis.

**Methodology:**

This study included 120 non-smoking males aged from 25 to 40 years (60 healthy patients and 60 with periodontitis). Each group was further subdivided into three subgroups according to serum 25-hydroxyvitamin D3 (25[OH]D3) levels: 0–10, 11–20, and 21–30 ng/mL. GCF samples were collected from the buccal sites of maxillary premolar or molar teeth. GCF osteocalcin and NTx levels were measured using enzyme-linked immunosorbent assay and statistically analyzed.

**Results:**

Total osteocalcin levels were significantly higher in healthy individuals than in those with periodontitis (p<0.001). The lowest osteocalcin levels were found in patients with periodontitis with 0–10 ng/mL vitamin D3 (p=0.001). Total NTx levels showed no significant differences between groups (p=0.148), but among healthy individuals, NTx decreased with increasing vitamin D3 (p=0.049).

**Conclusion:**

Patients with periodontitis and severe vitamin D3 deficiency showed the lowest GCF osteocalcin levels, suggesting that vitamin D3 deficiency may be associated with further alterations in local bone turnover activity in periodontal disease. Further studies are needed to explain these relationships.

ClinicalTrials.gov (Trial ID: NCT06871631)

## Graphical abstract

**Figure:**
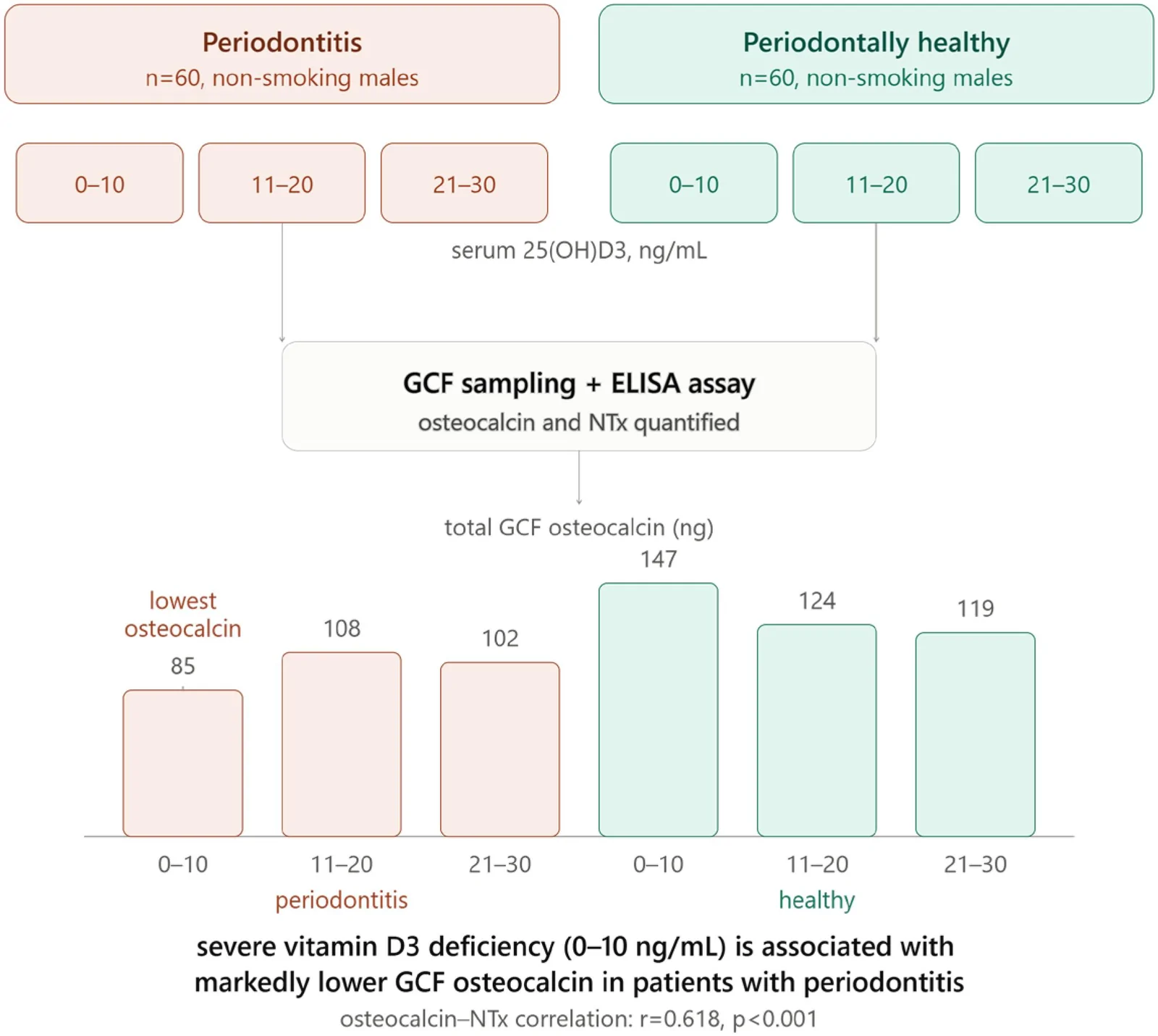

## Introduction

Periodontitis is a chronic inflammatory condition affecting the supporting structures of the teeth, leading to connective tissue breakdown and alveolar bone resorption.^1^ These pathological changes can be reflected locally in gingival crevicular fluid (GCF), which contains biochemical markers that offer valuable insights into the inflammatory and bone remodeling processes associated with disease activity and progression.^2^

Among these biomarkers, osteocalcin and N-terminal telopeptide (NTx) are particularly relevant. Osteocalcin is a small, non-collagenous protein synthesized by osteoblasts that serves as a marker of bone formation.^3^ Its synthesis is directly stimulated by 1,25-dihydroxyvitamin D3 [1,25(OH)_2_D3], the biologically active form of vitamin D3, which underscores the link between systemic vitamin D3 status and bone turnover activity.^4^ NTx, on the other hand, is released during the degradation of type I collagen and reflects bone resorption dynamics. As a bone-specific marker that is less influenced by soft tissue collagen turnover, it is also suitable for site-specific assessment in GCF.^5^

Vitamin D3 plays a key role in bone metabolism by regulating calcium-phosphate homeostasis and stimulating osteoblastic activity.^6^ To accurately assess vitamin D3 status, serum 25(OH)D3 concentration should be measured as it represents the major circulating and most stable form of vitamin D3, reflecting dietary intake and endogenous synthesis via sunlight exposure.^7^ In addition to its established role in systemic bone homeostasis, accumulating evidence suggests that vitamin D3 deficiency may contribute to periodontal tissue destruction by its effects on immune modulation, inflammatory response, and matrix degradation pathways.^8-10^

Given the potential influence of vitamin D3 on local bone turnover and periodontal tissue dynamics, this study aimed to evaluate the association between serum 25(OH)D3 levels and GCF levels of osteocalcin and NTx in periodontally healthy patients and those with periodontitis. Accordingly, this study hypothesized that serum vitamin D3 status is associated with GCF bone turnover markers in periodontal health and disease.

## Methodology

### Study population

This cross-sectional observational study was conducted in accordance with the Declaration of Helsinki and approved by the Medical Research Ethics Committee of Ondokuz Mayis University, Samsun, Turkey (Protocol No: OMUKAEK-2017/60). This study was registered at ClinicalTrials.gov (Trial ID: NCT06871631) and supported by the Ondokuz Mayis University Scientific Research Projects Commission (Project No: PYO.DIS.1904.17.019). Written informed consent was obtained from all participants before enrollment.

A total of 120 non-smoking male participants aged from 25 to 40 years were recruited from January 2018 to February 2019 from the Periodontology and Endocrinology Clinics of Ondokuz Mayis University. Only non-smoking males aged from 25 to 40 years were included to reduce biological variability and minimize potential confounding factors, including hormonal fluctuations associated with sex differences and age-related changes that may influence vitamin D3 metabolism and periodontal parameters. Data regarding sunlight exposure, dietary vitamin D3 intake, and body mass index were not specifically recorded during recruitment. Participants had neither received periodontal treatment within the previous six months nor had systemic conditions such as diabetes, cardiovascular disease, autoimmune disorders, chronic kidney disease, or osteoporosis (which could influence periodontal status or vitamin D3 levels). Serum 25(OH)D3 levels were determined from venous blood samples using liquid chromatography–tandem mass spectrometry (LC–MS/MS; München, Germany) in the Endocrinology Clinic laboratory.

Vitamin D3 status was classified according to the Institute of Medicine guidelines, which define serum 25(OH)D3 levels below 20 ng/mL as deficient.^11^ Although serum 25(OH)D3 levels above 30 ng/mL are generally considered optimal, concentrations from 21 to 30 ng/mL have been described as representing relative vitamin D insufficiency rather than severe deficiency.^12^ Therefore, participants within this range were designated as the reference control group to enable comparisons within the available study population. This selection reflects a pragmatic design choice, considering the generally low serum vitamin D3 levels in the regional population and the limited availability of individuals with serum 25(OH)D3 levels ≥30 ng/mL during the recruitment period. Accordingly, Groups 3 and 6 were used as internal reference controls for patients with periodontitis and healthy individuals, respectively. Participants were categorized into six groups based on their periodontal status and serum 25(OH)D3 levels:

**Group 1:** Patients with periodontitis with 25(OH)D3 levels of 0-10 ng/mL

**Group 2:** Patients with periodontitis with 25(OH)D3 levels of 11-20 ng/mL

**Group 3:** Patients with periodontitis with 25(OH)D3 levels of 21-30 ng/mL

**Group 4:** Periodontally healthy with 25(OH)D3 levels of 0-10 ng/mL

**Group 5:** Periodontally healthy with 25(OH)D3 levels of 11-20 ng/mL

**Group 6:** Periodontally healthy with 25(OH)D3 levels of 21-30 ng/mL

Each group consisted of 20 participants, totaling 120 volunteers equally distributed across the six groups.

### Clinical examination

A comprehensive periodontal examination was performed using a manual periodontal probe (UNC-15, Hu-Friedy, Chicago, IL, USA). Clinical parameters included plaque index,^13^ gingival index,^14^ probing pocket depth (PPD), clinical attachment level (CAL), bleeding on probing (BOP), and a radiographic evaluation of alveolar bone loss.

Participants were classified as having generalized Stage III, Grade B periodontitis according to the 2017 classification system established by the European Federation of Periodontology and the World Workshop.^15^ Diagnosis was based on interdental CAL ≥5 mm, PPD ≥6 mm in at least two non-adjacent teeth, radiographic bone loss extending to the middle third of the root or beyond in ≥30% of the teeth, and BOP ≥10%. They had lost ≤4 teeth due to periodontitis. CAL from conditions unrelated to periodontitis (such as endodontic lesions, cervical root caries, trauma-induced gingival recession, or distal bone loss adjacent to third molar extraction sites) was considered non-indicative of periodontitis. Such cases were excluded from analyses.

Participants in the periodontally healthy group showed PPD ≤3 mm, BOP <10%, and no detectable CAL at interproximal sites. No radiographic bone loss was observed. These findings were consistent with an intact periodontium and the absence of clinical signs of periodontitis.

### Collection of samples

GCF samples were collected one day after the clinical and radiographic examinations to minimize the risk of qualitative or quantitative changes in periodontal status. Standardized paper strips (Periopaper^®^, Ora Flow Inc., Amityville, NY, USA; 2 mm×14 mm) were used for GCF collection.

Prior to sampling, the site was isolated with cotton rolls to prevent salivary contamination. Any supragingival plaque was gently removed using a sterile curette without contacting the gingiva. The area was dried using sterile cotton pellets and air spray. Paper strips were gently inserted approximately 2–3 mm into the gingival sulcus of maxillary premolar or molar teeth, avoiding mechanical trauma. The strips were left in place for 30 seconds to absorb the fluid.^16^ GCF was collected from a single site for each individual. In participants with periodontitis, GCF was collected from the site with the deepest probing depth. The buccal surface was selected in periodontally healthy participants. To standardize the procedure and reduce variability, all samples were obtained from the buccal surface of a maxillary premolar or molar, selected for their accessibility, reduced risk of contamination, and consistent GCF yield. Strips contaminated with saliva or blood were discarded.

GCF volumes were measured using the Periotron^®^ 8000 (Pro Flow Inc., Amityville, NY, USA), which quantifies fluid volume based on electrical capacitance changes. The strips were then transferred to sterile Eppendorf tubes (Eppendorf AG, Hamburg, Germany) and stored at –80°C until biochemical analysis. Prior to the assays, the strips were eluted in the assay buffer provided by the ELISA kit and centrifuged, and the resulting supernatants were used for analysis according to the manufacturer's instructions. Osteocalcin and NTx were detectable in all GCF samples.

A comprehensive periodontal examination and GCF sampling were carried out by a single calibrated examiner (B.Ç.), ensuring procedural consistency across all participants.

### Biochemical analysis

GCF samples were centrifuged at 10,000 g for 15 minutes. The resulting supernatants were collected. The samples were thoroughly mixed using a vortex mixer before analysis.

Total amounts of osteocalcin and NTx in GCF were determined using commercially available ELISA kits: human osteocalcin/bone gla protein ELISA kit (Cat No. YLA1183HU) and human cross-linked n-telopeptide of type I collagen ELISA kit (Cat No. YLA0594HU) (both from Shanghai YL Biotech Co. Ltd., Shanghai, China). The analyses were performed using the sandwich ELISA method (following the manufacturer's protocols) in the Medical Biochemistry Laboratory of the Ondokuz Mayis University Faculty of Medicine.

Standard curves were constructed using serial dilutions to calculate total analyte levels in each sample. High-concentration samples were re-assayed for confirmation. For osteocalcin, the inter- and intra-assay coefficient of variation (CV) totaled <10% and <8%, respectively, with a 0.5–150 ng/mL detection range and a 0.26 ng/mL sensitivity. For NTx, the inter- and intra-assay CV totaled <10% and <8%, respectively, with a 0.5–200 nmol/L detection range and a 0.26 nmol/L sensitivity.

Although total and concentration values could be calculated by factoring in GCF volume, only total amounts were used in the statistical analyses due to their proposed biological relevance in reflecting site-specific periodontal bone turnover, as suggested in previous studies.^17,18^

### Statistical analysis

A priori power analysis was performed on G*Power (version 3.1.9.7) to determine the minimum sample size required to detect a clinically relevant difference in GCF osteocalcin levels between groups. The calculation assumed a 22.44 minimal relevant difference, a 18.7 standard deviation, a 0.05 significance level (*α*), and a 0.83 desired power. Groups were statistically compared via one-way ANOVA. Based on these parameters, the required sample size totaled 20 participants per group (which was obtained in this study).

Statistical analyses were performed on SPSS (version 21.0; IBM Corp., Armonk, NY, USA). Data distribution normality was assessed by the Shapiro–Wilk test. Quantitative data are shown as mean ± standard deviation when normally distributed and as median (min–max) otherwise. Comparisons between two independent groups were conducted using the Student's *t*- or–Whitney U tests depending on data distribution. For more than two groups, one-way ANOVA or the Kruskal–Wallis H test was applied accordingly. Post-hoc comparisons were performed using Tukey's honestly significant difference or Tamhane's T2 following ANOVA. Dunn–Bonferroni correction followed Kruskal–Wallis testing. Correlation between osteocalcin and NTx levels was evaluated using Spearman's rank correlation analysis. A p-value <0.05 was considered statistically significant.

## Results

### Demographic and clinical parameters

This study included 120 male participants, of whom 60 were periodontally healthy and 60 had periodontitis (classified based on clinical and radiographic assessments). Each periodontal status group was further stratified into three subgroups based on serum vitamin D3 levels (0–10, 11–20, and 21–30 ng/mL), with 20 participants in each subgroup, resulting in six groups. Descriptive data on participants’ demographics, serum vitamin D3 levels, and periodontal clinical parameters are shown in Table 1.

**Table 1:** Demographic characteristics and clinical periodontal parameters across study groups, stratified by periodontal status and serum 25(OH)D levels.

	G1 (P 0–10)	G2 (P 11–20)	G3 (P 21–30)	G4 (H 0–10)	G5 (H 11–20)	G6 (H 21–30)	p-value
	(n= 20)	(n= 20)	(n= 20)	(n= 20)	(n= 20)	(n= 20)	
Age (years)	33.2 ± 4.1^b^	34 ± 3.9^b^	33.5 ± 3.8^b^	31 ± 3.2^a^	30.5 ± 3.4^a^	30.1 ± 3.1^a^	**<0.001**
Serum 25(OH)D (ng/mL)	8.2 ± 1.1^a^	16.7 ± 2.3^b^	25.5 ± 1.7^c^	7.9 ± 1.2^a^	15.9 ± 2.5^b^	24.8 ± 2^c^	**<0.001**
BOP (%)	62.5 ± 11^b^	60.1 ± 12^b^	57.3 ± 10^b^	7.8 ± 4^a^	8.2 ± 5^a^	6.9 ± 3^a^	**<0.001**
CAL (mm)	3.9 (3.2–4.4)^b^	3.4 (2.9–3.9)^b^	3.1 (2.6–3.5)^b^	0 (0–0)^a^	0 (0–0)^a^	0 (0–0)^a^	**<0.001**
PPD (mm)	4.1 (3.5–4.7)^b^	3.9 (3.1–4.5)^b^	3.5 (2.9–4.2)^b^	2.3 (2–2.7)^a^	2.2 (2–2.5)^a^	2.1 (1.9–2.4)^a^	**<0.001**
PI	2.4 (2–2.7)^b^	2.2 (1.8–2.5)^b^	2 (1.6–2.3)^b^	0.5 (0.4–0.6)^a^	0.6 (0.5–0.7)^a^	0.5 (0.4–0.6)^a^	**<0.001**
GI	2.1 (1.7–2.4)^b^	1.9 (1.5–2.3)^b^	1.8 (1.4–2.1)^b^	0.4 (0.3–0.5)^a^	0.5 (0.3–0.6)^a^	0.4 (0.3–0.5)^a^	**<0.001**

*Note*: Data are shown as means ± SD or medians (min–max). A p-value <0.05 was considered statistically significant. Different lowercase letters within rows indicate significant differences between groups. Statistical comparisons were performed using one-way ANOVA with Tukey's post-hoc test for normally distributed variables and Kruskal–Wallis test with Dunn–Bonferroni correction for non-parametric variables.

*Abbreviations*: PPD, probing pocket depth; CAL, clinical attachment level; PI, plaque index; GI, gingival index; 25(OH)D, 25-hydroxyvitamin D.

*G1–G3 (P)*: Periodontitis groups with serum vitamin D levels totaling 0–10, 11–20, and 21–30 ng/mL, respectively.

*G4–G6 (H)*: Periodontally healthy groups with serum vitamin D levels totaling 0–10, 11–20, and 21–30 ng/mL, respectively.

All participants were non-smoking males aged from 25 to 40 years. A statistically significant difference in age was observed between the groups, with patients with periodontitis being slightly older on average (p<0.001). Clinical periodontal parameters (including CAL, PPD, plaque index, and gingival index) were significantly higher in the periodontitis groups than in healthy ones (p<0.001). No attachment loss or pathological pocket depth was detected in the healthy groups.

### GCF osteocalcin and NTx levels

Table 2 shows the total osteocalcin and NTx levels in GCF for periodontitis and healthy groups regardless of vitamin D3 status. Osteocalcin levels were significantly lower in the periodontitis group than in the healthy group (p<0.001), whereas the difference in NTx levels was not statistically significant (p=0.148).

**Table 2:** Comparison of total osteocalcin and NTx levels in gingival crevicular fluid between the periodontitis and healthy groups.

	Periodontitis (n = 60)	Healthy (n = 60)	p-value
Osteocalcin total (ng)	97.6 (49.8–135.5)	124.4 (58.2–259.3)	**<0.001**
NTx total (nmol)	223.7 (120.4–419.8)	234.7 (84.4–490.5)	0.148

*Note*: Data are shown as medians (min–max). Values represent total amounts per sampled site (ng or nmol). Statistical comparisons were made using the Mann–Whitney U test. A *p*-value < 0.05 was considered statistically significant.

*Abbreviations:* NTx, N-terminal telopeptide

Table 3 shows the total osteocalcin and NTx levels across six subgroups (categorized by periodontal status and serum vitamin D3 levels). In the periodontitis groups (G1–G3), osteocalcin levels were significantly lower in G1 than in G2 and G3 (p=0.001). No significant differences in NTx levels were observed between the periodontitis subgroups (p=0.121). In the periodontally healthy groups (G4–G6), NTx levels were significantly lower in G6 than in G4 and G5 (p=0.049), whereas osteocalcin levels showed no significant differences between groups (p=0.384).

**Table 3:** Total osteocalcin and NTx levels across study groups stratified by periodontal status and serum 25(OH)D levels.

	G1 (P 0–10)	G2 (P 11–20)	G3 (P 21–30)	p-value	G4 (H 0–10)	G5 (H 11–20)	G6 (H 21–30)	p-value
	(n=20)	(n=20)	(n=20)		(n=20)	(n=20)	(n=20)	
Osteocalcin total (ng)	85.31 ± 18.19^a^	107.75 ± 19.2^b^	101.59 ± 17.29^b^	**0.001**	147.33 (58.24-259.31)	123.7 (70.01-152.89)	118.89 (89.33-227.65)	0.384
NTx total (nmol)	205.4 (120.41-419.77)	226.51 (123.24-373.94)	230.48 (154.67-344.98)	0.121	275.29 (94.03-490.5)^a^	249.9 (134.1-365.89)^a^	210.87 (84.43-461.42)^b^	**0.049**

*Note:* Data are shown as means ± SD or median (min–max). Values represent total amounts per sampled site (ng or nmol). Statistical comparisons were performed using one-way ANOVA, followed by Tukey's honestly significant difference post-hoc test for normally distributed data and by the Kruskal–Wallis test for non-parametric data with Dunn–Bonferroni correction. A p-value < 0.05 was considered statistically significant. Different lowercase letters within rows indicate significant differences between groups.

*Abbreviations:* NTx, N-terminal telopeptide

*G1–G3 (P):* Periodontitis groups with serum vitamin D levels of 0–10, 11–20, and 21–30 ng/mL, respectively.

*G4–G6 (H):* Periodontally healthy groups with serum vitamin D levels of 0–10, 11–20, and 21–30 ng/mL, respectively.

Additionally, a statistically significant and moderately strong positive correlation was observed between total osteocalcin and total NTx levels (p<0.001), (Figure 1).

**Figure 1:**
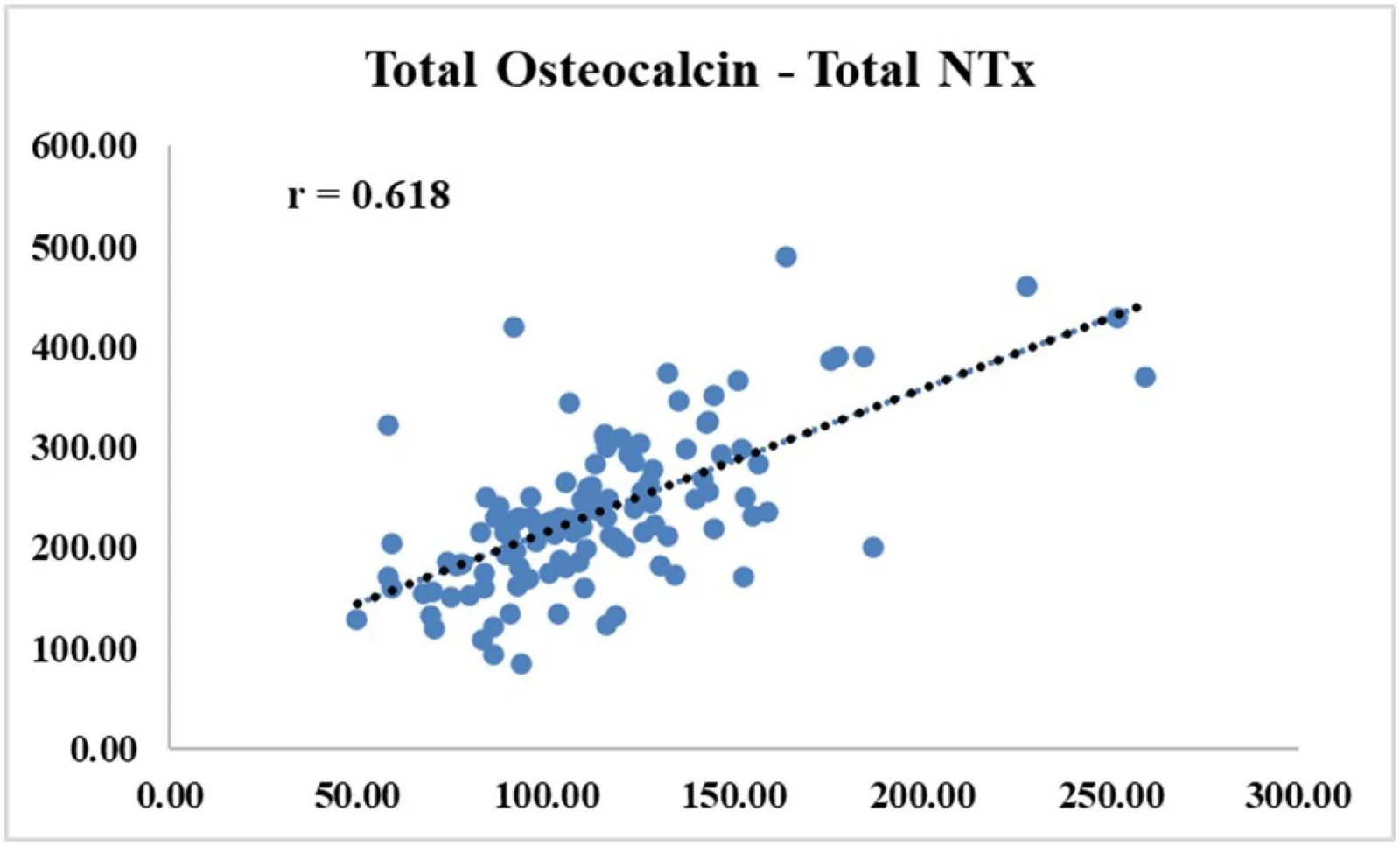
Graphs of Spearman correlation analysis results of total osteocalcin and total NTx levels

## Discussion

This cross-sectional study evaluated the association between serum vitamin D3 levels and GCF bone turnover markers in periodontally healthy patients and individuals with periodontitis. The most notable finding was that patients with periodontitis and severe vitamin D3 deficiency (0–10 ng/mL) showed markedly lower GCF osteocalcin levels, suggesting that severe systemic vitamin D3 deficiency may be associated with altered local bone turnover activity in the presence of periodontal inflammation.

GCF, an inflammatory exudate originating from serum, transudates into the gingival sulcus via the inflamed periodontal tissues. Its biochemical composition reflects the local inflammatory and bone remodeling activity at specific periodontal sites.^19^ Unlike whole-mouth saliva analysis, GCF collection enables site-specific assessment of periodontal tissue status.^20^ Previous studies have shown that GCF contains a variety of biomarkers, including host-derived cytokines, enzymes, matrix degradation products, inflammatory mediators, and bone turnover markers such as osteocalcin and NTx, which may reflect local bone metabolism.^17,19,21^ GCF sampling using filter paper strips via the intracrevicular method (as in this study) offers a minimally invasive and reliable approach for obtaining these biomarkers for biochemical analysis.^16^

This study adds further evidence regarding the association between systemic vitamin D3 status and local periodontal bone turnover markers in gingival crevicular fluid. While numerous studies have evaluated various inflammatory biomarkers such as calprotectin in GCF (reflecting neutrophil activity and general inflammatory burden), research focusing on bone turnover markers remains limited.^19,22,23,24^ Previous studies have reported fluctuations in GCF osteocalcin and NTx levels across periodontal conditions, suggesting altered local bone turnover dynamics in periodontitis.^17,18^ However, these studies ignored systemic vitamin D3 status as a potential modulating factor. Since osteocalcin synthesis is directly stimulated by the active form of vitamin D3, 1,25(OH)_2_D3, systemic deficiency may further impair osteoblastic function. Our findings provide additional evidence that severe vitamin D3 deficiency is associated with lower GCF osteocalcin levels in patients with periodontitis, suggesting altered local bone turnover dynamics.

In contrast to osteocalcin, total NTx levels, which reflect bone resorption activity, did not differ significantly between periodontitis and healthy groups in our study. This finding is consistent with some previous reports on variable or inconsistent changes in GCF NTx levels depending on periodontal disease activity.^17,18^ Interestingly, we observed a modest but statistically significant decrease in NTx levels with increasing vitamin D3 status (p=0.049) in periodontally healthy individuals. Vitamin D3 has been suggested to influence bone resorption via regulatory pathways in osteoclast activity, which may partially explain the observed differences in NTx levels.^25^ As NTx reflects collagen degradation associated with osteoclastic bone resorption, the observed decrease in NTx levels with increasing vitamin D3 status in the healthy individuals in our study may suggest a potential association between vitamin D3 status and local bone resorption dynamics. Nevertheless, the magnitude of this association was limited, and no significant differences were detected between health and disease groups. Previous studies have shown that periodontal tissue destruction and bone turnover biomarker release may exhibit temporal and site-specific variability during periodontitis.^5,19,26^ Accordingly, the absence of a statistically significant difference in NTx levels between periodontitis and healthy groups in this study may have been influenced by variations in disease activity, sampling timing, and individual biological responses affecting GCF NTx levels.

Several observational studies have shown that low serum vitamin D3 levels may be associated with increased susceptibility to periodontal disease, potentially due to its effects on immune modulation and bone metabolism.^8,9^ However, most previous research has primarily focused on systemic measurements and clinical periodontal parameters, with limited investigation into local biochemical markers reflecting bone metabolism within the periodontium.^8,27^ This study contributes to this area by exploring the relationship between systemic vitamin D3 status and site-specific GCF levels of osteocalcin and NTx, providing additional observational data on the potential interplay between systemic deficiency and local bone turnover activity.

The observed association between severe vitamin D3 deficiency and reduced GCF osteocalcin levels raises the question of whether improving systemic vitamin D status could enhance local bone formation activity during periodontal therapy. Although some clinical trials have suggested potential benefits of adjunctive vitamin D3 supplementation in improving periodontal treatment outcomes, current evidence remains limited and inconsistent.^10,28^ Further interventional studies are needed to explain the therapeutic implications of vitamin D3 supplementation in periodontal management.

The findings of this study should be interpreted in light of several study-specific considerations related to participant selection, vitamin D3 classification, and potential confounding factors. The inclusion of only non-smoking males aged from 25 to 40 years was intended to reduce biological variability and minimize potential confounding effects, as serum 25(OH)D3 levels vary with age and sex-related hormonal factors.^29^ Moreover, serum vitamin D3 levels are influenced by multiple external and individual factors, including seasonal variation, sunlight exposure, dietary intake, and body mass index.^30,31^ Toy et al. have shown that the impact of seasonal variation can be minimized by distributing participants evenly throughout the year during recruitment.^32^ However, this study was unable to specifically standardize such factors due to the extended recruitment period required for a relatively homogeneous study population. These unmeasured lifestyle-related variables may have partially contributed to the intergroup differences in serum vitamin D3 levels, possibly influencing the observed associations with periodontal biomarkers. Consistent with these considerations, the distribution of serum vitamin D3 levels in this study population also reflected region-specific patterns. Participants with serum 25(OH)D3 levels above 30 ng/mL were insufficiently represented in the study population. Therefore, individuals with 21–30 ng/mL levels were considered as the reference group to enable meaningful comparisons within the available sample. It should be noted that this range was pragmatically used to compare osteocalcin and NTx levels across groups with different vitamin D3 statuses rather than interpreted as reflecting optimal or sufficient vitamin D3 status. Moreover, previous studies in the same geographic region (including those from the Black Sea region of Türkiye) have consistently reported generally low serum 25(OH)D3 levels across healthy and diseased individuals, supporting the limited availability of individuals with higher vitamin D3 levels in this population.^8,33^ Accordingly, the findings should be interpreted within the context of these methodological and population-specific characteristics.

This study has several limitations that should be acknowledged. Its cross-sectional design precludes causal inference. Participants with serum 25(OH)D3 levels above 30 ng/mL were unavailable. The inclusion of only non-smoking males aged from 25 to 40 years, while intended to reduce biological variability and minimize potential confounding effects, limits the generalizability of the findings to broader populations. Furthermore, proinflammatory biomarkers and vitamin D3 supplementation were ignored. Potential confounding factors affecting vitamin D3 status (including seasonal variation, sunlight exposure, dietary intake, and body mass index) were also ignored. These limitations should be considered when interpreting the findings in this study, particularly regarding the generalizability of its results.

## Conclusion

In this study, patients with periodontitis showed significantly lower GCF osteocalcin levels than periodontally healthy individuals. Notably, the lowest osteocalcin levels were observed in patients with periodontitis with severe vitamin D3 deficiency (0–10 ng/mL), suggesting that marked vitamin D3 deficiency may be associated with altered bone remodeling activity in the presence of periodontal inflammation. No significant difference was detected in total NTx levels between health and disease groups, although a decrease in NTx was observed with increasing vitamin D3 levels among healthy individuals. These findings highlight a potential link between systemic vitamin D3 status and local bone turnover activity, particularly under severe deficiency, warranting further longitudinal studies to explain these associations.

## Data Availability

The datasets generated during and/or analyzed in this study are available from the corresponding author on reasonable request.
